# Supplementary material for: The interactome of intact mitochondria by cross-linking mass spectrometry provides evidence for coexisting respiratory supercomplexes
Source: Mol Cell Proteomics. 2017 Dec 8;17(2):216–32. doi: 10.1074/mcp.RA117.000470 (PMC5795388; doi:10.1074/mcp.RA117.000470)
Supplement: Supplemental Data [file supp_RA117.000470_133922_0_supp_23978_fzffwf.zip › spectra_annotation/mito_DR_spectra_annotation/279-1-4-1-6-1.pdf]

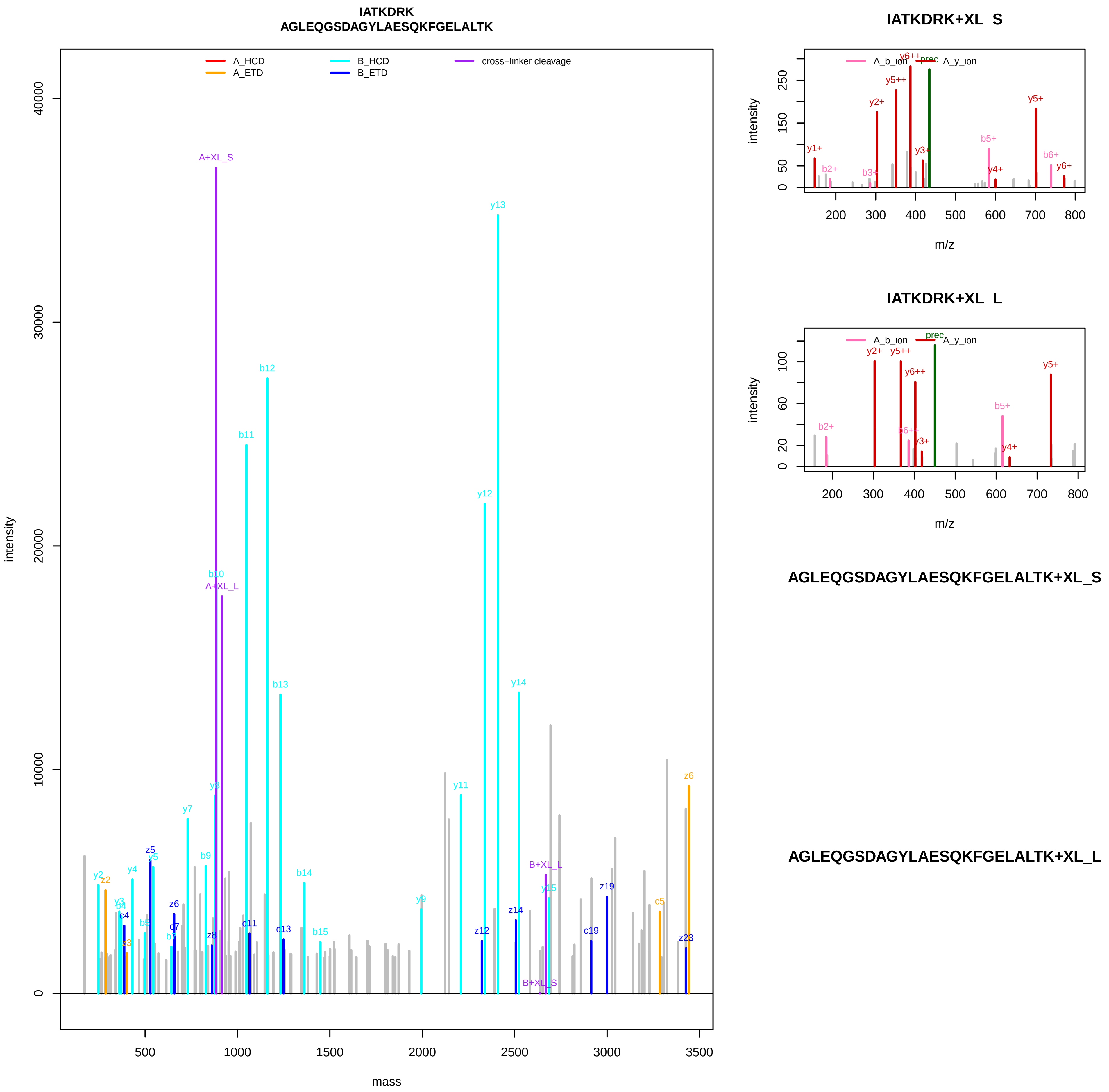
IATKDRK
AGLEQGSDAGYLAESQKFGELALTK
A_HCD
A_ETD
B_HCD
B_ETD
cross−linker cleavage
A+XL_S
A+XL_L
B+XL_L
B+XL_S
y13
b12
b11
y12
b10
b13
y14
y11
z6
c5
y8
y7
z5
y5
b9
y4
y2
z2
b14
z19
y15
y9
c4
b4
y3
z14
c11
c7
b7
z8
c13
z12
c19
z23
b15
z3
b5
intensity
mass
0
10000
20000
30000
40000
500
1000
1500
2000
2500
3000
3500
IATKDRK+XL_S
A_b_ion
A_y_ion
prec
y6++
y5++
y2+
y5+
y1+
b2+
b3+
y3+
b5+
y4+
b6+
y6+
intensity
m/z
0
50
150
250
200
300
400
500
600
700
800
IATKDRK+XL_L
y2+
y5++
y6++
y5+
b2+
b5+
b6++
y3+
y4+
0
20
60
100
AGLEQGSDAGYLAESQKFGELALTK+XL_S
AGLEQGSDAGYLAESQKFGELALTK+XL_L
